# Neuroimaging analyses from a randomized, controlled study to evaluate plasma exchange with albumin replacement in mild-to-moderate Alzheimer’s disease: additional results from the AMBAR study

**DOI:** 10.1007/s00259-022-05915-5

**Published:** 2022-07-22

**Authors:** Gemma Cuberas-Borrós, Isabel Roca, Joan Castell-Conesa, Laura Núñez, Mercè Boada, Oscar L. López, Carlota Grifols, Miquel Barceló, Deborah Pareto, Antonio Páez

**Affiliations:** 1 Research & Innovation Unit, Althaia Xarxa Assistencial Universitària de Manresa, Carrer Dr. Joan Soler 1-3, 08242 Manresa, Spain; 2 Department of Nuclear Medicine, Hospital Universitari Vall d’Hebrón, Universitat Autònoma de Barcelona, Barcelona, Spain; 3 Alzheimer’s Research Group, Grifols, Barcelona, Spain; 4 Ace Alzheimer Center Barcelona - Universitat Internacional de Catalunya, Barcelona, Spain; 5 Centro de Investigación Biomédica en Red de Enfermedades Neurodegenerativas (CIBERNED), Instituto de Salud Carlos III, Madrid, Spain; 6 Departments of Neurology and Psychiatry, University of Pittsburgh School of Medicine, Pittsburgh, PA USA; 7 Radiology Department (IDI), Hospital Universitari Vall d’Hebron, Barcelona, Spain

**Keywords:** Alzheimer’s disease, Plasmapheresis, Plasma exchange, Albumin, Intravenous immunoglobulin

## Abstract

**Purpose:**

This study was designed to detect structural and functional brain changes in Alzheimer’s disease (AD) patients treated with therapeutic plasma exchange (PE) with albumin replacement, as part of the recent AMBAR phase 2b/3 clinical trial.

**Methods:**

Mild-to-moderate AD patients were randomized into four arms: three arms receiving PE with albumin (one with low-dose albumin, and two with low/high doses of albumin alternated with IVIG), and a placebo (sham PE) arm. All arms underwent 6 weeks of weekly conventional PE followed by 12 months of monthly low-volume PE. Magnetic resonance imaging (MRI) volumetric analyses and regional and statistical parametric mapping (SPM) analysis on ^18^F-fluorodeoxyglucose positron emission tomography (^18^FDG-PET) were performed.

**Results:**

MRI analyses (*n* = 198 patients) of selected subcortical structures showed fewer volume changes from baseline to final visit in the high albumin + IVIG treatment group (*p* < 0.05 in 3 structures vs. 4 to 9 in other groups). The high albumin + IVIG group showed no statistically significant reduction of right hippocampus. SPM ^18^FDG-PET analyses (*n* = 213 patients) showed a worsening of metabolic activity in the specific areas affected in AD (posterior cingulate, precuneus, and parieto-temporal regions). The high-albumin + IVIG treatment group showed the greatest metabolic stability over the course of the study, i.e., the smallest percent decline in metabolism (MaskAD), and least progression of defect compared to placebo.

**Conclusions:**

PE with albumin replacement was associated with fewer deleterious changes in subcortical structures and less metabolic decline compared to the typical of the progression of AD. This effect was more marked in the group treated with high albumin + IVIG.

**Trial registration:**

(AMBAR trial registration: EudraCT#: 2011–001,598-25; ClinicalTrials.gov ID: NCT01561053).

**Supplementary Information:**

The online version contains supplementary material available at 10.1007/s00259-022-05915-5.

## Introduction

Dementia is a growing public health concern worldwide. It is estimated that the number of people living with dementia will be 131.5 million by 2050 [1]. Alzheimer’s disease (AD) accounts for 60–80% of these cases of dementia [2]. The behavioral symptoms of AD include cognitive impairment, memory loss, depression, and deficits in communication and spatial perception [3]. Microscopically, AD is characterized by neurofibrillary tangles (intracellular) and amyloid plaques (extracellular) made up of phosphorylated tau proteins and amyloid β peptides (Aβ), respectively [4, 5]. Both of these defects are thought to contribute to neuronal cell death.

In the efforts to attack the underlying disease process in AD, one of the primary targets has been Aβ. Accumulation of Aβ in brain tissue and vasculature is thought to be an important part of the AD process [6]. Although the therapeutic strategy of targeting Aβ to improve function in AD patients has been until recently largely unsuccessful [7–9], a potential clinical benefit associated with reduction of Aβ plaques using anti-Aβ antibodies is currently contributing to the understanding of Aβ pathophysiology [10–13]. Furthermore, additional interest in Aβ has been piqued by the observation that sequestration of Aβ in the circulation resulted in a decrease in Aβ in the cerebrospinal fluid (CSF) [14]. This led to the “sink hypothesis” that removal of Aβ from the circulation could lead to a decrease in Aβ in the CSF and the brain.

Given that Aβ in blood is bound to albumin (1:1) [15], it was proposed that plasma exchange (PE) with albumin replacement could be used to remove Aβ from the peripheral circulation. Removal of the patient’s plasma would result in removal of albumin-bound Aβ. PE would be followed by replacement of the lost volume along with exogenous albumin to avoid hypovolemia and maintain osmotic pressure [16]. This could result in further sequestration of Aβ through binding to the fresh Aβ-free [17] albumin. Additional PE could produce further reductions in CSF/brain Aβ through equilibration with plasma Aβ [18].

The neuroimaging studies described in this paper were part of a multicenter, randomized, controlled, phase 2b/3 study to evaluate the efficacy and safety of PE with albumin replacement with or without alternated intravenous immunoglobulin (IVIG) in AD patients (AMBAR Trial: EudraCT#: 2011–001,598-25; ClinicalTrials.gov ID: NCT01561053). In a previous phase 2 study [19], positive clinical changes associated with PE treatment were found to parallel neuroimaging data [20]. Similarly, the previously published clinical results from the AMBAR study showed that PE treatment slowed the decline in cognitive, functional, and neuropsychological outcomes in AD patients, while improving their quality of life [21–23].

## Material and methods

### Objective and study design

The neuroimaging objective of the AMBAR study was to detect functional and structural changes in the brains of mild-to-moderate AD patients treated with PE with therapeutic albumin replacement in comparison with placebo (sham-PE) patients. These outcomes were assessed using magnetic resonance imaging (MRI) and ^18^F-fluorodeoxyglucose positron emission tomography (^18^FDG-PET).

The AMBAR trial enrolled patients at 41 sites: 19 in Spain and 22 in the USA. This study was conducted in compliance all applicable regulatory guidelines and the International Council for Harmonization of Technical Requirements for Pharmaceuticals for Human Use (ICH) Good Clinical Practice (GCP). Each patient and caregiver, family member, or legal representative provided informed consent prior to enrollment. The Institutional Review Boards (IRBs) or Ethics Committees from the sites and the Health Authorities from the countries where the study was performed approved the protocol, the informed consent form, and the patient information sheets.

### Patient population

In the AMBAR trial, 322 patients were evaluable and 232 patients (72%) completed the study. Reasons for discontinuation are summarized in Supplemental Table S1 for placebo and active treatment groups. Further details have been previously reported elsewhere [23].

Inclusion criteria were men and women between 55 and 85 years of age with a diagnosis of probable AD dementia according to the National Institute of Neurological and Communication Disorders and Stroke and the Alzheimer’s Disease and Related Disorders Association (NINCDS-ADRDA) criteria [24], with baseline Mini-Mental State Exam (MMSE) score from 18 to 26 [25], and on a stable dose of AChEIs and/or memantine. Exclusion criteria included cerebrovascular disease, IgA deficiency, low hemoglobin, high plasma creatinine, uncontrolled hypertension, liver disease, heart diseases, any condition in which the treatment was contraindicated or not feasible, and fewer than 6 years of education. Full details of eligibility criteria are available elsewhere [22, 23].

The population was 54% female with an average age of 69.0 ± 7.7 (mean ± SD) years. The patients were equally divided in terms of mild (*n* = 161, baseline Mini-Mental State Examination [MMSE] score: 22–26) and moderate AD (*n* = 161, baseline MMSE score: 18–21). Patients were free of cerebrovascular disease and had been diagnosed with AD since 2.4 ± 2.4 years based on criteria from the NINCDS-ADRDA criteria [24]. These patients had a baseline MMSE score of 21.3 ± 2.6 [25] and were being treated with acetylcholinesterase inhibitors and/or memantine at a stable dose. Further details of demographic and clinical characteristics of patients have been previously published [23] and summarized for placebo and active treatment groups in Supplemental Table S1.

### Treatment groups

Eligible patients were randomly assigned one of the four groups (1:1:1:1), three PE-treated groups and one placebo group. During intensive treatment phase, the three treatment groups received weekly conventional therapeutic PE (TPE) with albumin (Albutein® 5%, Grifols) replacement, through peripheral or central venous access, for 6 weeks. This was followed by a 12-month maintenance period with monthly low-volume PE (LVPE) during which three different treatment modalities were administered: one group received LVPE with low-dose (20 g; 100 mL) 20% albumin (Albutein® 20%, Grifols); one group received low-dose albumin alternated with low-dose (10 g; 200 mL) IVIG (Flebogamma® 5% DIF, Grifols) every 4 months; and one group received high-dose (40 g; 200 mL) 20% albumin alternated with high-dose (20 g; 400 mL) IVIG every 4 months. The placebo group received a simulated non-invasive PE (sham) procedure that did not involve any fluid transfer [22, 23]. Placebo patients were subjected to the same procedures and evaluations as PE-treated patients. The patients, caregivers, and neuroimaging evaluators were blinded as to the therapy received.

### Image acquisition

MRI and ^18^FDG-PET measures were taken at baseline (month 0; M0), the intermediate visit after the intensive treatment phase (month 2; M2), month 9 (M9), and the final visit (month 14; M14). MRI scans were also performed at month 6 (M6).

Images were acquired at different imaging sites, MRI with a 1.5 T magnet scanner, and 18F-FDG-PET with PET/CT scanners. Each participating imaging site acquired and reconstructed the data according to a standardized protocol following the Alzheimer’s Disease Neuroimaging Initiative (ADNI) recommendations [26].

#### MRI acquisition

Patients were asked to lie in supine position, the center of the coil was aligned with the line of the eyes, they were instructed not to move, and the head was gently stabilized with cushions. Whole-brain T1-weighted images of 1-mm isotropic resolution were obtained at isocenter using the magnetization-prepared rapid gradient-echo (MP-RAGE) pulse sequences included in a vendor-supplied package, and using the same acquisition protocol (TR = 2300 ms, TE = 2.98 ms, field of view 256 mm, and 192 sagittal partitions, voxel size = 1 × 1 × 1 mm).

#### ^18^FDG-PET acquisition

Patients who were claustrophobic or non-cooperative, those without suitable venous access to administer the tracer, and those taking corticosteroids at the time of the test were excluded. Patients fasted for at least 4 h prior to the procedure to provide optimal brain ^18^FDG uptake that was not influenced by increased blood glucose levels.

Before the injection of ^18^FDG, blood glucose levels were checked to assure that they were lower than 160 mg/dL. Patients were placed in a quiet, dimly lit room several minutes before ^18^FDG administration, and during the ^18^FDG uptake phase (at least 20 min), talking, reading, or listening were avoided. Sedation of patients was not allowed.

A dose of 185 ± 18 MBq (5 ± 0.5 mCi) ^18^FDG was administered, and 30 min after injection, the images were acquired. Acquisition of CT images was carried out first. Next, ^18^FDG-PET images were acquired in 3D mode, with an acquisition duration of 15 min, matrix size 128 × 128, slice thickness (Z) of 2.0 mm, and pixel size (X, Y) of 2.0 mm × 2.0 mm. The images were reconstructed with iterative algorithm (15 subsets and 4 iterations).

The images were corrected for attenuation with the CT scan, and were reviewed to assess possible artifacts and movement. Eventually the reconstructed images were reoriented on the orbitomeatal axis.

### Individual image analysis

Longitudinal analysis of the ^18^FDG-PET and MRI scans was limited to patients who completed all study visits and had analyzable scans at all visits.

#### MRI quantification

FIRST software from the Oxford Centre for Functional Magnetic Resonance Imaging of the Brain (FMRIB) Software Library (FSL) (Oxford, UK) [27] and SPM8 software (Welcome Trust Centre for Neuroimaging, London, UK) running on MATLAB (MATLAB R2012b, MathWorks, Natick, MA, USA) were used to compute brain volumetry.

FIRST, a fully automated method, was applied to the MPRAGE images to derive subcortical volumes. MRI images were reoriented according to the anatomic FSL atlas, using the fslorient2std tool. Next, the neck region was extracted with the robustfov tool and ran FIRST to determine the volume of the right and left thalamus, putamen, caudate, pallidum, hippocampus, amygdala, accumbens area, and brainstem plus the fourth ventricle.

In addition, the following cortical volumetries were analyzed: total intracranial volume, overall gray matter (GM), white matter (WM), and cerebrospinal fluid (CSF) volumes, together with fractions of all these measures with respect to the total intracranial volume (TIV). MRI scans were normalized and segmented by using the default unified segmentation methods of SPM8 to obtain GM, WM, and CSF segmented masks. Gray matter and white matter volumes were obtained and segmented, as well as cerebrospinal fluid (CSF) for each patient, and the TIV was calculated as the sum of gray, white, and CSF volumes, allowing generation of the corresponding gray and white matter relative to the total intracranial volume.

#### ^18^FDG-PET quantification

In the first part of this study, a quantitative approach based on voxel-by-voxel analysis was performed to assess the global metabolic activity of the entire brain (by creating parametric images of the metabolic defect pattern and its follow-up), as well as individual brain region analysis to identify individual cerebral metabolic abnormalities.

Image pre-processing was carried out by using SPM8. To start, ^18^FDG-PET images were spatially normalized onto the Montreal Neurological Institute (MNI) space, intensity normalized to the cerebellum mean (as this is a large and stable region and it is known to be minimally affected by age-related changes) [28–30] and spatially smoothed using an isotropic Gaussian kernel with 8 mm FWHM to increase the signal-to-noise ratio and to account for the subtle variations in anatomical structures.

The individual parametric images of the metabolic defect pattern were computed using the intensity normalized and smoothed image sets comparing them to an in-house and tracer-, and age-matched normal-reference brain ^18^FDG-PET template (in MNI space) based on 48 healthy elderly controls recruited for other unicentric study (see Supplemental Table S2 for details). All subjects in the control population were scanned using same scanner, with the same methodology and protocols as in the present study. This approach compares voxel-by-voxel each patient’s ^18^FDG-PET scan with those of the healthy controls. This comparison allowed the detection of metabolic defects based on the Z-score for an individual voxel and comparison to the mean and standard deviation of the reference template. A voxel that, when compared to a control voxel at the same anatomic position in MNI space in the normal-reference brain ^18^FDG-PET template, had a value that is lower than the average by at least two standard deviations was considered a defect. Based on the Z-score for an individual voxel and comparison to the mean and the standard deviation of the distribution templates created from the normal-reference template, each patient voxel was assigned a value based on a preset range (see Supplemental Table S3). These values were used to create an individual defect map. Each of these values was assigned a color indicating the severity of the lesion. For an individual subject, these parametric analyses could be compared over the course of the study. A particular voxel could be evaluated over time and evaluated as to whether it had improved (voxels that showed a defect at M0 and were not a defect at M14, or voxels that had an increase of Z-score of more than 0.5), maintained (defect or normal at M0 and the same at M14), or worsened (a voxel that was not a defect at M0, but was a defect at M14, or a voxel with a Z-score that worsened by 0.5) relative to the control database.

Finally, to minimize the effects of cerebral atrophy on the creation of the individual metabolic defect pattern, a correction mask was created from the gray matter probability maps of the same patient and applied to the result to remove those voxels which may correspond to areas of atrophy or increase/dilation of the ventricular cavities.

In order to allow comparisons between treatment groups and comparisons over time within treatment group, a regional analysis was performed by extracting the mean uptake values from the regions of the Automatic Anatomical Labeling (AAL) atlas [31], using an in-house algorithm, and statistical analyses were conducted. AAL atlas is a parcellation of a standardized MRI scan from the MNI and mean counts were extracted from its 116 mapped brain regions (see Supplemental Table S4).

When the ^18^FDG-PET scans were analyzed for the 116 brain areas (VOIs) using the AAL atlas, three parameters were measured: (1) metabolism intensity (mean counts per each region inside the cerebral atrophy correction mask computed from the normalized and smoothed image sets); (2) defect extension (% of abnormal voxels per each region inside the cerebral atrophy correction mask computed from the individual defect map), and (3) defect intensity (degree of defect per each region inside the cerebral atrophy correction mask computed from the individual defect map).

Finally, the baseline defect pattern was computed for all groups by computing the mean image from all individual defect maps.

### Voxel-based analysis with statistical parametric mapping (SPM)

Voxel-based analysis with SPM was used to establish the pattern of tissue abnormalities from the disease and to assess the global group effects of treatment over time.

The paired *t*-test implemented in SPM8 was applied considering a statistical threshold of *p* < 0.001 uncorrected and in relation to the unspecific hypometabolic voxels. Those detected voxels that survive a cluster thresholding using a minimum cluster size of k = 50 were considered.

SPM maps showing contrasts were superimposed onto a T1 template using MRIcron on 12 axial views covering the entire brain. To identify the location of the significant voxels obtained in the SPM analysis, the Wake Forest University (WFU) PickAtlas tool [32] was used.

For each treatment group, the evolution of k-extent (metabolism loss over time (change from baseline)/longitudinal analysis: M2 vs M0; M9 vs M0, and M14 vs M0) was shown in maximal intensity projection (MIP) view. The measurement of defect extent and defect intensity took into consideration all the voxels within the VOI.

The SPM maps showing metabolic decline from M0 to M14 in the placebo group allowed identification of the regions of the brain involved in the natural progression of AD. These data were used to create the Alzheimer’s Disease mask (MaskAD). The MaskAD when applied to extract the mean uptake from the scans of each individual in the treatment groups and allowed the determination of the metabolic defect intensity in the treatment groups compared to placebo.

### Statistics

Descriptive statistics were calculated for all measured variables (mean, median, standard deviation, 25th percentile, 75th percentile, minimum, and maximum). Comparisons between treatment groups and longitudinal comparisons (M0, M2, M6, M9, and M14) were made using analysis of variance (ANOVA). Pairwise comparisons of different time points within a treatment group were made using Student’s *t*-test with a significance level set at *p* < 0.05. Statistical analyses were performed using SAS software (version 9.2, Cary, NC, USA).

## Results

### Samples for image processing

Of the total study population of 322 evaluable patients from AMBAR study, ^18^FDG-PET scans were missing or unable to be analyzed for 13 patients at baseline (M0). At final visit (M14), 224 images were available. For longitudinal SPM analysis, only patients that completed all study visits and without images unable to be processed could be included leaving 213 patients for ^18^FDG-PET longitudinal analysis. For MRI, 55 patients had missing or unreadable scans at M0. At M14, 198 images were available. This was the database available for analysis of VOI.

Most patients had both ^18^FDG-PET and MRI scans available: *n* = 257 at M0 (representing 83.2% of ^18^FDG-PET and 96.3% of MRI), and *n* = 183 at M14 (representing 81.7% of ^18^FDG-PET and 92.4% of MRI).

### Brain volumetry analysis by MRI

No changes were reported between treatment groups at each cross-sectional time point and in all subcortical and cortical areas and globally there was a loss in volume throughout the 14-month period.

There was a significant volume loss of GM (in absolute values and fraction) in all treatment groups and placebo from M0 to M14. WM in absolute values were not significantly decreased in any treatment group and placebo group, and WM fraction was only reduced in the placebo group, but not in any active treatment groups. On the contrary, significant volume loss of CSF (in absolute values and fraction) was observed in all active treatment groups, but not in the placebo group from baseline to final visit (M14).

When volumetry for subcortical structures was analyzed longitudinally (from M0 to M14), the placebo group showed a significant volumetry loss in 4 areas (hippocampus [L, R] and accumbens [L, R]). For the low albumin group, significant decreases in volume were shown in 9 areas (caudate [L, R], hippocampus [L, R], accumbens [L, R], amygdala [L], putamen [R], and pallidum [R]). The low albumin + IVIG group showed a significant volumetry loss in 8 areas (thalamus-proper [L, R], caudate [L, R], accumbens [L, R], putamen [L], and hippocampus [R]). For the high albumin + IVIG, there was a significant volumetry loss in 3 areas: amygdala [L], caudate [R], and accumbens [R]. Therefore, nucleus accumbens [R] was significantly reduced in all treatment groups (active and sham), whereas caudate [R] was significantly reduced in all active treatment groups, but not in placebo group. Pallidum [L] and brain stem 4th ventricle areas were not significantly decreased for all active treatment and placebo groups. All groups except high albumin + IVIG showed a statistically significant reduction of hippocampus [R].

Globally, fewer statistically significant changes in volume were observed over time in the high albumin + IVIG treatment group (*p* < 0.05 in 3 structures vs. 4 to 9 in other groups) (Table 1).

**Table 1:** Subcortical structures with significant (*p* < 0.05) structural (volumetric) changes measured by MRI between baseline and month 14 in Alzheimer’s disease patients treated with plasma exchange (PE) with low- or high-dose albumin with or without intravenous immunoglobulin (IVIG)

Placebo	PE-treatment		
	Low Albumin	Low Albumin + IVIG	High Albumin + IVIG
Accumbens [L, R]	Accumbens [L, R]	Accumbens [L, R]	Accumbens [R]
Hippocampus [L, R]	Amygdala [L]	Caudate [L, R]	Amygdala [L]
	Caudate [L, R]	Hippocampus [R]	Caudate [R]
	Hippocampus [L, R]	Putamen [L]	
	Pallidum [R]	Thalamus-proper [L, R]	
	Putamen [R]		
Total: 4/15 (26.7%)	Total: 9/15 (60%)	Total: 8/15 (53.3%)	Total: 3/15 (20%)

### Brain metabolism analysis by ^18^FDG-PET

**Fig. 1:**
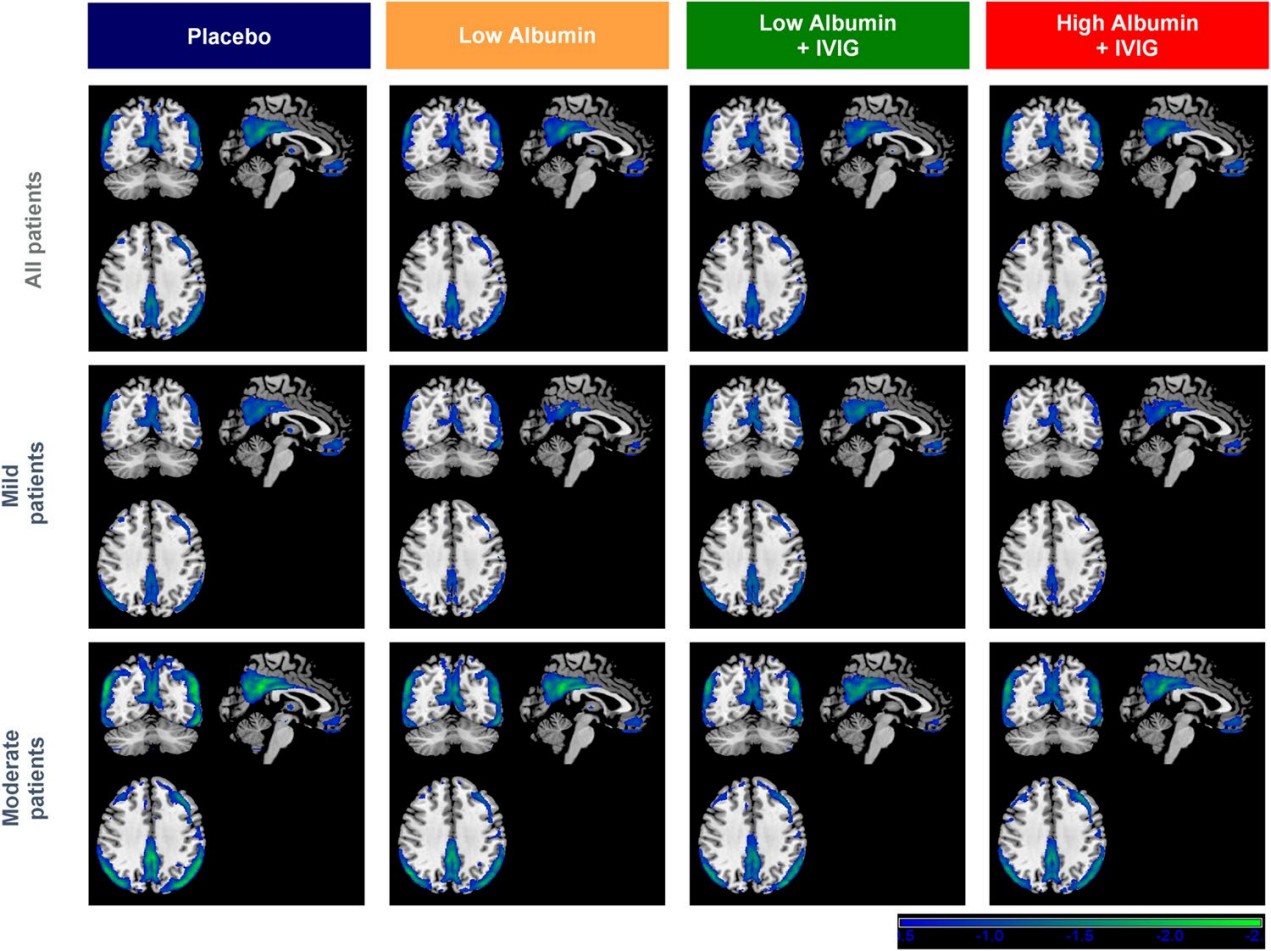
Defect pattern in ^18^F-flurodeoxyglucose positron emission tomography (^18^FDG-PET) analysis at baseline in Alzheimer’s disease patients treated with plasma exchange with low- or high-dose albumin with or without intravenous immunoglobulin (IVIG). Axial view

^18^FDG-PET analyses showed that the baseline defect pattern was consistent with AD (posterior cingulate, both parietal, temporal [slightly more extensive in the left hemisphere], and slight extension to the frontal) and was similar between all treatment groups. Similar defect patterns were obtained for mild and moderate AD patients. No differences were observed in the defect pattern between mild and moderate AD patients or between treatment groups. However, defect pattern in moderate AD patients was more intense (evidenced by the color of the defect, greener, which means more standard deviations in relation to the normality database) than in mild AD patients, and more extensive (Fig. 1).

Changes in metabolism intensity, defect extension, and defect intensity in all brain areas that had significantly changed between M0 and M14 are shown in Table 2. The high albumin + IVIG treatment group showed the lowest percentage of areas with significant changes in these parameters. Most of the changes in metabolic intensity were seen in the limbic structures, temporal and parietal cortex, and the cerebellum. The primary brain areas affected were the same as those seen when measuring metabolic intensity.

**Table 2:** Change in metabolism intensity, defect extension, and defect intensity in all brain areas (*N* = 116) expressed as a number and percentage of areas that significantly changed (*p* < 0.05) between baseline and month 14 in Alzheimer’s disease patients treated with plasma exchange (PE) with low- or high-dose albumin with or without intravenous immunoglobulin (IVIG)

	Placebo	PE-treatment		
		Low albumin	Low albumin + IVIG	High albumin + IVIG
Metabolism intensity	21 (18.1%)	58 (50.0%)	44 (37.9%)	29 (25.0%)
Defect extension	42 (36.2%)	63 (54.3%)	65 (56.0%)	36 (31.0%)
Defect intensity	56 (48.3%)	52 (44.8%)	58 (50.0%)	47 (40.5%)

### Parametric analysis of ^18^FDG-PET

**Fig. 2:**
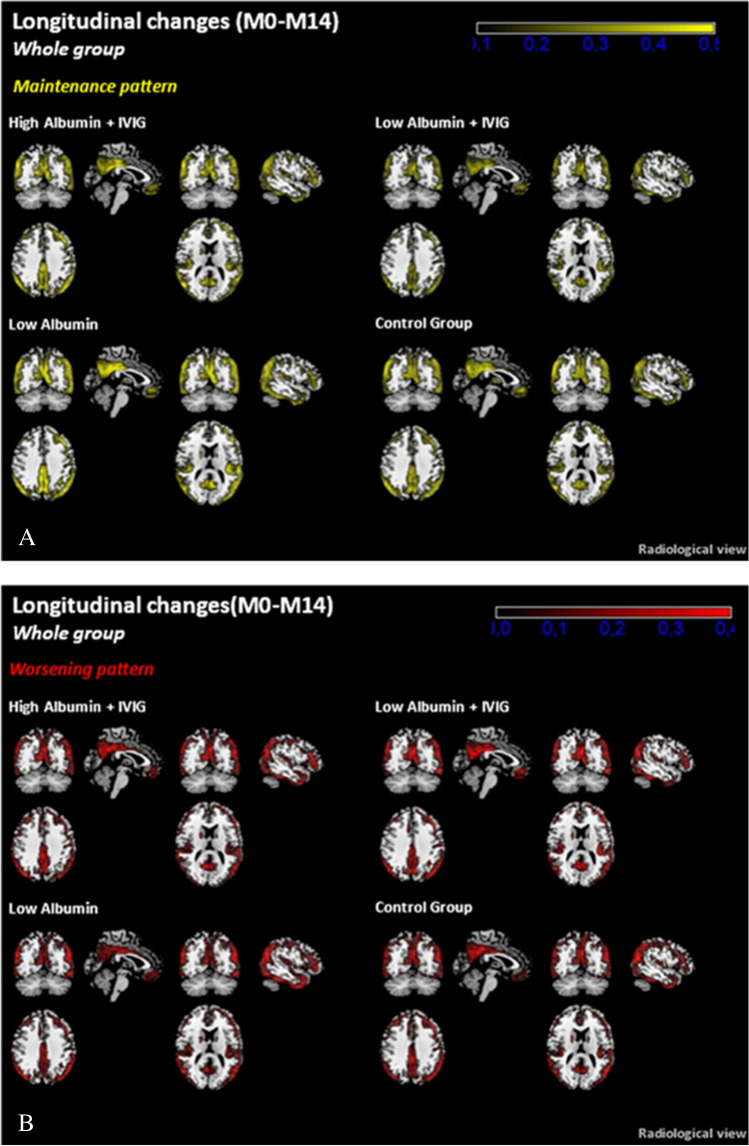
Triangulation view of the metabolic maintenance pattern (**A**: upper panel) and worsening pattern (**B**: lower panel) (parametric ^18^FDG-PET analysis) over the complete study period (baseline [M0] to month 14 [M14]) in Alzheimer’s disease patients treated with plasma exchange with low- or high-dose albumin with or without intravenous immunoglobulin (IVIG). All patients

The metabolic activity pattern is shown in Fig. 2. The areas that showed maintenance of metabolic activity were the posterior cingulum, precuneus, and parietal cortex extending into the temporal region (Fig. 2A). Analysis of the areas that showed worsening over the study period indicated that the areas affected were the posterior cingulum, precuneus, and parietal and temporal regions of the cortex (Fig. 2B). The high albumin + IVIG treatment group showed the greatest stability over the course of the study.

The percentage decline in metabolism (MaskAD) from M0 to M14 is shown in Table 3. When all the patient data was considered together, the least amount of decline was seen in the high albumin + IVIG group. By AD severity, decline during the study period was less in the mild AD group than in the moderate AD group. High albumin + IVIG had the greatest effect in the mild AD group. In the moderate AD group, the least decline was seen in the low albumin group. Table 3 shows that area of significant metabolic decline was less in the high albumin + IVIG group than in any of the other groups. This was true for the pooled data and when the patients were divided by AD severity. Greater loss of metabolism (increased MaskAD size) was seen in the placebo, low albumin, and low albumin + IVIG groups than in the high albumin + IVIG group. The high albumin + IVIG group showed the smallest MaskAD size in all three brain regions (temporal, parietal, and frontal).

**Table 3:** Percentage of metabolism loss in MaskAD between baseline and month 14 in Alzheimer’s disease patients treated with plasma exchange (PE) with low or high dose albumin with or without intravenous immunoglobulin (IVIG), stratified by disease severity

	Placebo	PE-treatment		
		Low albumin	Low albumin + IVIG	High albumin + IVIG
All patients	− 4.3	− 3.3	− 3.6	− 2.9
Mild AD	− 1.6	− 2.1	− 2.9	− 0.9
Moderate AD	− 6.6	− 3.6	− 4.3	− 4.2

**Fig. 3:**
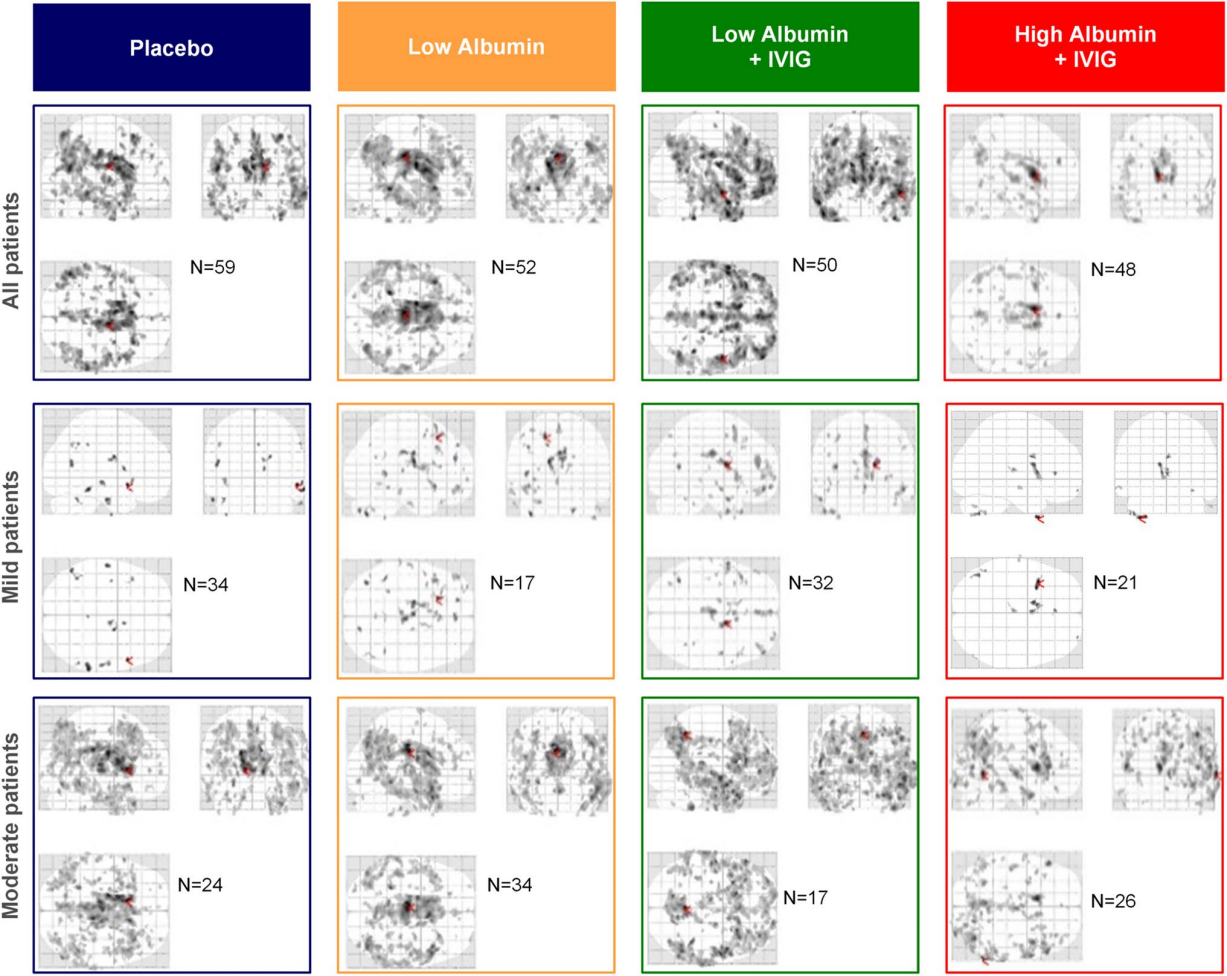
Maximum intensity projection (MIP) view of changes of SPM between baseline and month 14 in Alzheimer’s disease patients treated with plasma exchange with low- or high-dose albumin with or without intravenous immunoglobulin (IVIG), stratified by disease severity

Figure 3 shows the SPM analysis, where there was very little change in metabolism in mild AD patients treated with high albumin + IVIG. Some deterioration was seen in the other three treatment groups. As in the whole patient population, the effects of high albumin + IVIG were seen consistently across brain regions. There was essentially no change in metabolism in the parietal and frontal lobes over the course of the study in the high albumin + IVIG treatment group while metabolism declined in the other treatment groups.

Table 4 shows that area of significant metabolic decline (size of mask k-extent) in the SPM analysis was smallest in the high albumin + IVIG group. This was true for the pooled data and when the patients were divided by AD severity. Much greater loss of metabolism is reflected in the MIP view from the other treatment groups (Fig. 3).

**Table 4:** Metabolism loss in SPM ^18^FDG-PET in size of mask k-extent (in voxels) between baseline and month 14 in Alzheimer’s disease patients treated with plasma exchange (PE) with low- or high-dose albumin with or without intravenous immunoglobulin (IVIG), stratified by disease severity

	Placebo	PE-treatment		
		Low albumin	Low albumin + IVIG	High albumin + IVIG
All patients	83,998	118,074	143,269	30,822
Mild AD	2560	8050	9494	293
Moderate AD	101,251	91,978	104,177	49,706

The effects of treatment were more noticeable in the mild AD patient group. As shown in Supplemental Fig. S1A, at M14 the metabolic intensity decreased while the extension and intensity of defect slightly increased. As with the representative example of moderate AD patients (Supplemental Fig. S1B), the reduction of metabolic intensity at M14 was seen across all cerebral lobes, and more evident the extension and intensity of defect, which doubled.

## Discussion

Diagnosis and progression of AD are currently based on clinical observation, scoring on various assessments of cognitive function, and surrogate biochemical markers [33]. Most patients in the AMBAR study, who were enrolled based on the presence of the AD clinical syndrome, had baseline CSF Aβ_42_ levels ≤ 600 pg/mL [23]. This is in agreement with patient populations and methods of determination in other studies [23]. However, over the years, there has been increasing interest in the diagnostic utility of neuroimaging. This study explored ^18^FDG-PET and MRI results from the AMBAR study patients.

Among neuroimaging techniques, computed axial tomography was found to be useful in diagnosing some types of dementia but is of limited usefulness in discriminating AD [34]. Single positron emission computed tomography (SPECT) has also shown some utility in distinguishing some types of dementia [35, 36]. However, ^18^FDG-PET imaging is a more valuable tool in the assessment of patients with AD [37]. More recently, MRI has demonstrated utility due to a greater capability to distinguish anatomical structures in the brain and provide more precise images [38]. In this study ^18^FDG-PET and MRI scans performed on AD patients throughout the course of the study were compared with scans performed at baseline (M0) and scans from a population without AD. Moreover, SPM is considered the gold standard for dementia diagnosis [39]. With 213 patients analyzed (49 to 60 per treatment group), this study is one of the largest performed using this technique in a clinical trial.

MRI has been shown to detect structural changes in the brain associated with AD including cerebral atrophy and enlargement of the ventricles and sulci [40, 41]. Our brain volumetry studies showed that the high albumin + IVIG group had fewer structural changes from M0 to M14, including the stabilization of the hippocampus volume. Importantly, the hippocampus plays a key role in developing new memories [42] and is one of the first areas in the brain affected by AD [43].

^18^FDG-PET analyses of the effects of PE on AD using these scans showed that baseline pattern of defect was consistent with AD [44–47] and similar between all treatment groups. After treatment, metabolic deficits were still seen, but in fewer brain areas and were less extensive in the high albumin + IVIG treatment group than in the other treatment groups or the placebo group. This was particularly evidenced in defect extension and defect intensity, the parameters directly related to neuronal damage rather than metabolic intensity. These effects were observed primarily in the brain regions most affected by AD (posterior cingulate, precuneus and parieto-temporal lobes) [48–50].

When looking at the longitudinal metabolic decline in all brain areas (MaskAD or number of voxels showing a metabolic decline at the end of the study compared to baseline), mild AD patients showed a pattern of metabolic decline over time slower than moderate AD patients, as expected. However, the increase in intensity and extension of defect were not significantly different between any of the treatment groups and placebo. This could be explained by the fact that minimal changes in the high albumin + IVIG group were balanced by the extensive pattern of defect in the two low albumin groups (with or without IVIG), which had change patterns similar to placebo. This suggests that the mild AD patients’ group was more stable over time, regardless of the treatment arm.

Interestingly, the clinical results showed that PE-treated mild AD patients scored better in verbal memory and language fluency tests (particularly in Rey auditory verbal learning test [RAVLT], semantic verbal fluency [SVF], and phonetic verbal fluency PVF]) [21]. This paralleled the reduced metabolic deficit observed in the posterior cingulate gyrus and parietal lobe, where damage is suggestive of AD pathology in patients with language deficits [44, 48]. Moreover, ^18^FDG uptake in the posterior cingulate-precuneus or in language-related Brodmann areas has been shown to correlate the main indices of RAVLT in patients with mild cognitive impairment (MCI) [51] and in AD [52]. These results are important because PE could stabilize hypometabolism or hypoperfusion in mild AD. These defects are predictors of a rapid progression to more severe AD [53]. More specifically, the angular region, along with the posterior cingulate, are regions that present greater progression of metabolic loss. This finding is in agreement with the findings of Rosselli et al. [54] in which they demonstrated with structural connectivity techniques the integrative role of the angular region in linguistic functions.

In moderate AD patients, there was a significant difference in the extension of defect pattern between high albumin + IVIG and the rest of treatment arms. Low albumin (regardless of IVIG) presented extension defect patterns similar to placebo, whereas high albumin + IVIG group had less loss of metabolism by intensity and extension than the rest. This suggests that the higher dose of albumin was necessary to achieve the neuroprotective effects.

Previously published data from pilot and phase 2 studies showed that the positive clinical effects of PE in AD patients [19, 55] were correlated with positive neuroimaging data [20, 55]. Taking these findings to a further step, dose separation and separate mild-to-moderate AD patients were assessed in this phase 2b/3 study. Clinical results showed that some of the positive cognitive and behavioral effects of PE appeared to be more pronounced in moderate AD patients [23], while memory, language, and processing speed abilities appeared to be more pronounced in mild AD patients [21]. In the present neuroimaging analyses, some positive effects of PE treatment were seen in the mild AD group, while others were seen in the moderate AD group, particularly with high albumin + IVIG*.*

A potential limitation of this study is that some of the analyses such as metabolism intensity, pattern of defect, and defect intensity are semi-quantitative, which could conceal some of the complexity of AD etiopathology. Additionally, a study period longer than 14 months may be necessary to fully assess whether the treatment has a meaningful impact on the course of AD in these patients. Determining the exact relationship between the effects of PE on brain structure and function (as measured by ^18^FDG-PET and MRI) and cognitive and behavioral effects will require additional investigation.

In conclusion, PE treatment in AD patients was associated with fewer deleterious changes in selected subcortical structures and less metabolic decline in the specific areas affected in AD than the typical progression of the disease, as observed in the placebo group. These effects were more marked in the group treated with high albumin + IVIG compared to the low albumin groups (with or without IVIG) and in moderate AD patients. Mild AD patients seemed metabolically more stable over time, regardless of the treatment arm.

## Supplementary Information

Below is the link to the electronic supplementary material.

Supplementary file1 (DOCX 1063 KB)

## Data Availability

Data reported in this manuscript are available within the article and/or its supplementary materials. Additional data from the AMBAR study (EudraCT#: 2011–001,598-25; ClinicalTrials.gov ID: NCT01561053) are available from the corresponding author upon reasonable request.
