# Supplementary material for: Neuroimaging analyses from a randomized, controlled study to evaluate plasma exchange with albumin replacement in mild-to-moderate Alzheimer’s disease: additional results from the AMBAR study
Source: Eur J Nucl Med Mol Imaging. 2022 Jul 22;49(13):4589–600. doi: 10.1007/s00259-022-05915-5 (PMC9606044; doi:10.1007/s00259-022-05915-5)
Supplement: Supplementary file 1 — Supplementary file1 (DOCX 1063 KB) [file 259_2022_5915_MOESM1_ESM.docx]

Supplemental Table S1. Summary of demographic and clinical characteristics of patients at baseline and discontinuations

| **Characteristic** | **Placebo** | **Treatment modalities** | | |
| --- | --- | --- | --- | --- |
|  |  | **Low albumin** | **Low albumin  + IVIG** | **High albumin  + IVIG** |
| All patient population, N | 80 | 78 | 86 | 78 |
| Moderate AD (MMSE 18-21), N | 36 | 46 | 37 | 36 |
| Mild AD (MMSE 22-26), N | 44 | 32 | 49 | 42 |
| MMSE score, mean ± SD | 21.7 ± 2.6 | 21.2 ± 2.4 | 22.1 ± 2.6 | 21.4 ± 2.6 |
| Age (yrs), mean ± SD | 68.4 ± 8.4 | 68.5 ± 7.5 | 69.5 ± 6.9 | 69.5 ± 7.9 |
| Female sex, % | 45.0 | 55.1 | 55.8 | 60.3 |
| BMI (Kg/m^2^), mean ± SD | 26.8 ± 4.3 | 27.0 ± 4.8 | 27.1 ± 4.0 | 26.5 ± 5.0 |
| Time since diagnosis of AD (yrs), mean ± SD | 2.5 ± 2.3 | 2.2 ± 2.4 | 2.5 ± 2.3 | 2.4 ± 2.6 |
| ApoƐ4 carriers, % | 44.2 | 63.5 | 48.2 | 44.6 |
| CSF Aβ_42_pg/mL, mean ± SD | 602 ± 268 | 560 ± 206 | 602 ± 287 | 620 ± 295 |
| Completed the study, N | 64 | 61 | 56 | 51 |
| Reasons for discontinuation |  |  |  |  |
| Adverse event, n | 1 | 6 | 15 | 9 |
| Consent withdrawal, n | 12 | 4 | 11 | 10 |
| Other reasons, n | 3 | 7 | 4 | 8 |

Supplemental Table S2. Characteristics of the healthy subjects used as a reference population (n= 48) and their source study

| Characteristics | Result |
| --- | --- |
| Institution | Hospital Universitari Vall d’Hebron (Barcelona, Spain) |
| Informed consent signed | Yes |
| Female / male (n) | 33 / 15 |
| Age, years (mean ± SD) | 65.6 ± 6.6 |
| Education | < 6 years: n= 2  ≥ 6 years: n= 31  Not available: n= 15 |
| Cognitive normality evaluation | Physician exploration  MMSE (>28 in all patients)  ^18^FDG-PET and MRI |
| Exclusion criteria | Diabetes  Medical history of neurologic disorders or mental health,  Other medical condition not controlled in anamnesis |

^18^FDG-PET: ^18^F-flurodeoxyglucose positron emission tomography; MRI: magnetic resonance imaging; MMSE: Mini-Mental State Examination

Supplemental Table S3. Parametric values, based on the Z-score range for an individual voxel and comparison to the mean and standard deviation templates created from the normal brain atlas

| Value | Z-score |
| --- | --- |
| 3 | ≥4 |
| 2 | ≥ 3 and <4 |
| 1 | ≥ 2 and <3 |
| 0 | ≥ −2 and <2 |
| −1 | ≥ −2 and > −3 |
| −2 | ≥ −3 and > −4 |
| −3 | ≤ −4 |

Supplemental Table S4. Automated Anatomic Labeling (AAL) brain regions by lobe.

| **Frontal** | **Occipital** | **Basal ganglia** |
| --- | --- | --- |
| Precentral_L, R | Calcarine_L, R | Caudate_L, R |
| Frontal_Sup_L, R | Cuneus_L, R | Putamen_L, R |
| Frontal_Sup_Orb_L, R | Lingual_L, R | Pallidum_L, R |
| Frontal_Mid_L, R | Occipital_Sup_L, R | Thalamus_L, R |
| Frontal_Mid_Orb_L, R | Occipital_Mid_L, R | **Cerebellum** |
| Frontal_Inf_Oper_L, R | Occipital_Inf_L, R | Cerebelum_Crus1_L, R |
| Frontal_Inf_Tri_L, R | Fusiform_L, R | Cerebelum_Crus2_L, R |
| Frontal_Inf_Orb_R | **Temporal** | Cerebelum_3_L, R |
| Rolandic_Oper_L, R | Heschl_L, R | Cerebelum_4_5_L, R |
| Supp_Motor_Area_L, R | Temporal_Sup_L, R | Cerebelum_6_L, R |
| Olfactory_L, R | Temporal_Pole_Sup_L, R | Cerebelum_7b_L, R |
| Frontal_Sup_Medial_L, R | Temporal_Mid_L, R | Cerebelum_8_L, R |
| Frontal_Mid_Orb_L, R | Temporal_Pole_Mid_L, R | Cerebelum_9_L, R |
| Rectus_L, R | **Limbic structures** | Cerebelum_10_L, R |
| **Parietal** | Cingulum_Ant_L, R | Vermis_1_2 |
| Postcentral_L, R | Cingulum_Mid_L, R | Vermis_4_5 |
| Parietal_Sup_L, R | Cingulum_Post_L, R | Vermis_6 |
| Parietal_Inf_L, R | Hippocampus_L, R | Vermis_7 |
| Postcentral_R | ParaHippocampal_L, R | Vermis_8 |
| SupraMarginal_L, R | Amygdala_L, R | Vermis_9 |
| Angular_L, R | **Insula** | Vermis_10 |
| Precuneus_L, R | Insula_L, R |  |
| Paracentral_L, Robule_L, R |  |  |


**Supplemental Fig. S1**. Representative example of ^18^F-flurodeoxyglucose positron emission tomography (^18^FDG-PET) and magnetic resonance imaging (MRI) scans of Mild AD (A: upper panel) and moderate AD (B: lower panel) patients at baseline and at month 14 –Rows 3 and 4. Rows 1 and 3 show axial images from a normalized ^18^FDG-PET scan. Rows 2 and 4 show the parametric defect superimposed on axial images of the normalized T1-MRI scan
